# Theory of affective pragmatics under biolinguistics

**DOI:** 10.3389/fpsyg.2024.1404067

**Published:** 2024-10-01

**Authors:** Li Zhuo

**Affiliations:** ^1^School of Foreign Studies, Fuzhou University, Fuzhou, China; ^2^School of English and International Trade, Beijing Foreign Studies University, Beijing, China

**Keywords:** affective pragmatics, Darwinian biolinguistics, threshold, speech acts, emotional

## Abstract

This paper introduces a pioneering investigation into affective pragmatics through the perspective of Darwinian Biolinguistics, an interdisciplinary field at the nexus of biological and linguistic principles. Anchored in Darwin’s theory of evolution and the latest developments in neurobiology, this study delves into the influence of biological factors---especially those pertaining to the brain’s emotional processing on pragmatic communication. The research posits that human emotional responses, inherent in our biological constitution, profoundly influence the usage and interpretation of language in social interactions.

## 1 Introduction

Affective pragmatics represents an emerging interdisciplinary field at the intersection of linguistics, psychology, and neuroscience. This area of study delves into the significant influence emotions have on language use and interpersonal communication. It conceptualizes affect as the emotional attitude or psychological state linked to particular objects or phenomena, marked by feelings of attachment or detachment, and exhibiting a consistent orientation (Jacobson, 1988:18).

Pragmatics, with a history of less than a century, has long been dominated by rationalist theories, like the Cooperative Principle, Speech Act Theory, Relevance Theory, and the Neo-Gricean school. They concentrated primarily on deciphering meaning in information exchange, leaving less examined influence of emotional factors upon linguistic communication. Recent years, however, have witnessed the rising of interpersonal pragmatics which takes affect as a significant influencing factor in communication, yet the affect explored under interpersonal pragmatics is a mild one under rational control that leaves the atypical peripheral affective pragmatic behaviors out of its research list. In contrast, Theory of Affective Pragmatics, proposed by Scarantino (2017a) who is inspired by Darwin’s theory of evolution (Darwin, 1871) and Tomkins’ affect motivation theory (Tomkins, 1962), is the first one that aims to explore affect as the driving force behind speech acts and to infer pragmatic intentions through analysis of affect. By providing insights into “affective-cognitive-volitional (ACV) and affective-volitional-cognitive (AVC) types of speech acts” (Kopytko, 2004), it hopes to make up for the deficiency left by the rationalist approach. This paper consolidates the foundational concepts and examines the evolution of and the current debates over affect pragmatics to foster further academic exploration in this field, expecting to offer readers a panoramic view of the emerging field of study.

## 2 Literature review

Affect, integral to both humans and other animals, encapsulates an individual’s behavioral intentions and modes, as highlighted by Dewey (1895). Physiological needs, suggested by Tomkins (1962), often transform into emotions, which then serve as a driving force for behaviors. For instance, the physiological state of dehydration triggers an urgent sense of thirst, subsequently leading to the behavior of drinking (*ibid*). Building on this foundation, behaviorist psychology (e.g., Skinner, 1957), argues that psychological phenomena can essentially be represented through behaviors, with human actions being responses to external stimuli, leading to muscle contractions and gland secretions. This perspective posits that emotions, such as anger, can provoke aggressive behaviors, while unresolved negative emotions like anxiety may result in repetitive verbal behaviors (Skinner, 1957). Moreover, emotional development, transformation, and restraint in behaviors that provoke negative reactions are part of an individual’s growth, influenced by evaluative signal stimuli.

However, verbal behaviors may not always accurately reflect an individual’s true emotional state due to social conditioning and education that align expressions with mainstream values. It is in untrained nonverbal behaviors, such as micro-expressions, that one’s genuine emotions are more transparent. Along the line, affective pragmatics, emphasizes the importance of such nonverbal cues in discerning the truthfulness of speech. This understanding allows for the inference of behavioral tendencies and the preparation of corresponding strategies by interpreting emotional signals, as proposed by Darwin (1872) and Skinner (1957). Emotions can be categorized into positive and negative (Jacobson, 1988), further into specific types like joy, anger, and sadness, evolving from primitive to advanced stages, with the latter influenced by social development and encompassing moral, esthetic, and rational feelings.

Following Skinner (1957), cognitive behaviorism (e.g., Bandura, 2001), which merges cognitive psychology elements with the behaviorist approach, emerged as a dominant school of thought. By the late 20th century, both affect science and cognitive behaviorism had underscored the critical role of emotions in cognition, motivation, and human behavior. The debate between Zajonc (1980, 1984) and Lazarus (1991a, 1991b), focusing on the interplay between emotional and cognitive processes and questioning the necessity of cognitive appraisal for emotional responses, propelled the notion that emotions span a spectrum from minimally to highly cognitive. This spectrum suggests that basic emotions may conform more closely to Zajonc’s perspective, while complex emotions might be more in line with Lazarus’s conceptualization. Regardless, the influence of emotion on speech acts is undeniable and indispensable in pragmatic research. In the era marked by the affective turn, interpersonal pragmatics has embarked on exploring affective pragmatics. Nonetheless, this line of inquiry, particularly adopting Lazarus’s stance on affect (e.g., Langlotz and Locher, 2013, 2017), reveals limitations in addressing speech acts driven by strong emotions, indicating a significant potential for further application of affective pragmatics. Recently, there has been a resurgence of research in linguistic field. Some researchers (e.g., Vergis, 2023) have pointed out the vital role affect plays in shaping inferential processes, arguing that affect is integral to communication and essential for interpreting conversational nuances.

### 2.1 The development of affective pragmatics

Pragmatics, as Morris (1938) articulates, is the study of the interplay between individuals and symbols, intersecting with fields such as biology, psychology, and sociology. This discipline encompasses three primary branches: conversational pragmatics, functional pragmatics, and psychological pragmatics, with the foundational works of Austin (1962) and Searle (1969) in conversational pragmatics, often aligned with classical Gricean pragmatics—standing out as particularly influential (Horn, 1988). These pioneers laid the groundwork by delineating speech acts and advocating the Cooperative Principle, thus emphasizing the crucial role of meaning in effective communication.

Classical Gricean pragmatics advocates for adherence to the Cooperative Principle, which necessitates that communicators observe maxims of quantity, quality, relevance, and manner. However, real-world communication frequently exhibits deviations from these rational guidelines, presenting a challenge to Grice’s framework, especially in the theory of conversational implicature. Grice (1957) posited that the essence of understanding utterances lies in grasping the speaker’s intent, a process foundational to successful communication. Grice (1968) further pointed out that the foundation of successful communication lies in one party’s ability to clearly convey their mental intentions through speech, and the other party’s ability to accurately decode these intentions from the speech signals and respond appropriately. However, Austin’s classification of speech acts reveals a complexity of overlapping meanings and functions, highlighting the nuanced interplay between speaker intention and listener interpretation.

The intricate dance of communication also involves non-verbal elements such as sequence, stress, intonation, and tone, as Searle (1965) noted, which play a pivotal role in conveying intentions and eliciting appropriate responses under the Cooperative Principle. These speech elements primarily serve to express emotions, underscoring the affective dimensions of communication. Searle (1969) further explored how the efficacy of perlocutionary acts depend on the speaker’s ability to communicate intentions within the bounds of social conventions, which are influenced by esthetics, morality, and rationality.

Building on this, Searle (1975) in his exploration of ‘*Indirect Speech Acts*’, elucidated how communicators often relay implicit intentions through indirect means, enabling hearers to infer intentions beyond the literal meaning. This aspect of communication, where expressive speech acts serve multiple functions, resonates with the core principles of affective pragmatics and provides indirect evidence supporting the utility of affective illocutionary/perlocutionary mechanisms. Searle’s acknowledgment of the significance of emotions in communication and the multifunctionality of expressive acts aligns closely with affective pragmatics, enriching our understanding of the affective illocutionary/perlocutionary mechanism’s role in the nuanced landscape of human interaction.

In the realm of daily interactions, emotions play an indispensable role as pragmatic elements, significantly influencing the intentions behind speech acts. The introduction of the politeness principle by Leech (1983) aims to mitigate the limitations inherent in the cooperative principle, offering a nuanced understanding of speech acts motivated by emotions. This perspective marks a departure from traditional pragmatics, which primarily focuses on the logical dimensions of language, such as speech acts and implicatures, highlighting the complex interplay between volition, cognition, and affect within the human psyche. Each of these aspects contributes distinctly to the construction and interpretation of speech acts.

Emerging from this critique, relational pragmatics, as exemplified by Kopytko (2004), expands the horizon of pragmatic research beyond the rational framework. This approach identifies six distinct emotional pragmatic behaviors, delineated by the interplay among cognitive, affective, and volitional factors: cognitive-affective-volitional (CAV), cognitive-volitional-affective (CVA), volitional-cognitive-affective (VCA), volitional-affective-cognitive (VAC), affective-cognitive-volitional(ACV), and affective-volitional-cognitive(AVC) (*ibid*:543). The classification underscores a bifurcation where the initial three types, with cognition as the precursor to emotion, align with rational affective pragmatic behaviors. Conversely, the latter three categories, where emotion precedes cognition, epitomize emotional affective pragmatic behaviors.

**Figure 1:**
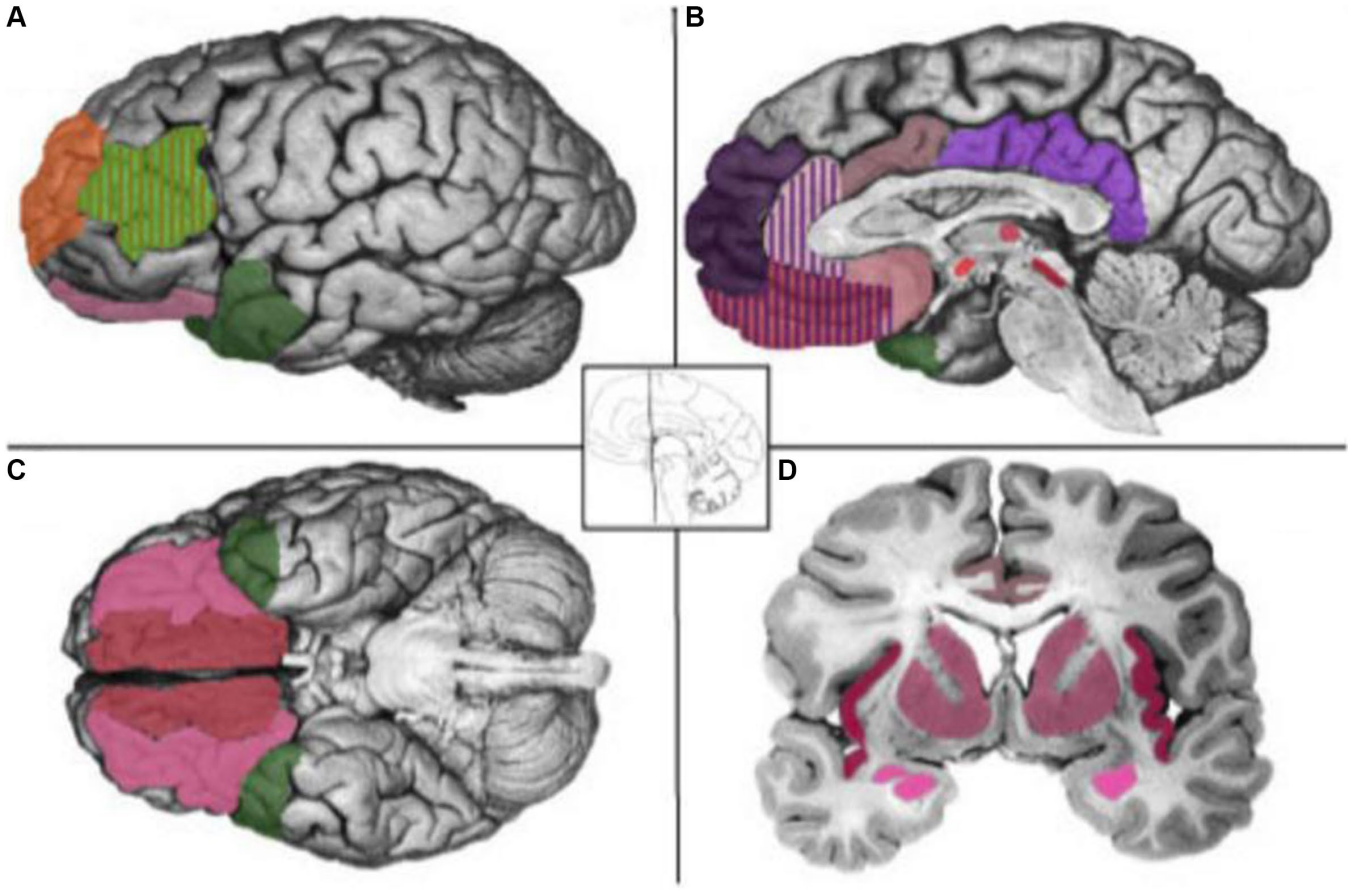
Psychological Constructionist Hypotheses of Brain–Emotion Correspondence **(A)** Lateral view. **(B)** Sagital view at the midline. **(C)** Ventral view. **(D)** Coronal view. Reproduced with permission from Lindquist et al. (2012).

Further enriching this discourse, neurophysiological research has elucidated the correlation between specific emotions and brain regions, such as the amygdala’s association with fear, the anterior cingulate gyrus with sadness, the orbitofrontal cortex with anger, and the insula with disgust (Figure 1) (Lindquist et al., 2012). This neurobiological grounding reveals how emotions elicit physiological neural responses, manifesting in facial expressions and speech acts. Such responses are indicative of the intricate relationship between physiological states, psychological experiences, and behavioral tendencies associated with each emotion. Building upon these insights, the field has witnessed the formal introduction of affective pragmatics, which delves into the operational mechanisms of emotional signals (Scarantino, 2017a). This development signifies a pivotal expansion in the study of pragmatics, embracing the profound impact of emotions on the intricacies of human communication.

Affective pragmatics, anchored in the theory of affect motivation, posits that verbal emotional signals are instrumental in deciphering an individual’s behavioral intentions and predispositions. This approach reconceptualizes human conversational behavior as a tripartite communicative act—signifying, acting, and accomplishing—mediated through verbal emotional cues (Scarantino, 2017a). While Searle (1969) delineates speech acts into assertive, directive, commissive, expressive, and declarative functions, affective pragmatics extends the expressive verbal acts to encompass asserting, committing, and directing functions as well (Scarantino, 2017a). It’s suggested that the expressive function in communication is not limited to verbal acts but also includes paralanguage, enriching the conveyance of emotions and intentions.

The alignment of verbal and nonverbal languages facilitates a unified transmission of emotions and intentions. Discrepancies between them, however, can lead to mixed messages, embodying conflicting emotions and intentions. For instance, directives can be conveyed both linguistically and paralinguistically, with apologies using paralinguistic behaviors to express shame and seek forgiveness. Similarly, assertive functions may indirectly communicate emotional evaluations through verbal or paralinguistic cues, indicating harm or danger through respective emotional expressions.

Scarantino (2017b) posits that emotional signals can substitute for verbal communication and enhance the accuracy of pragmatic inference, reducing errors. Recognizing paralinguistic cues, such as expressions of anger, can enable an accurate understanding of conversational functions and guard against deception by verbal signals (Scarantino, 2018). Despite the potential for feigning both verbal and paralinguistic acts, authentic emotional signals—often involuntarily expressed—distinguish themselves markedly from insincere manifestations. In everyday interactions, individuals may mask their anger to maintain a courteous image, yet involuntary facial expressions and paralinguistic behaviors may inadvertently reveal true emotions. Consequently, paralinguistic signals, such as facial expressions and posture, become critical in assessing emotions (Scarantino, 2019). In this way, affective pragmatics illustrates how communicators can achieve the objectives of speech acts through the recognition and interpretation of paralinguistic emotional cues, highlighting paths for both theoretical and applied research. Scarantino (2020) further explores how affect-driven actions enable individuals to engage in self-regulation by selectively focusing on specific emotions, thereby influencing others through emitted emotional signals to arouse their emotions.

However, the ambiguity inherent in emotional signals complicates speech acts and poses challenges for research. Scarantino et al. (2022) discuss how collective verbal aggression can stem from strong emotions, indicating that emotions can amplify and influence the collective. This insight underpins the theory that collective actions, sometimes unconscious, are motivated by affect. For instance, Blitvich (2022) identifies emotion as a primary catalyst for online verbal aggression, challenging the traditional pragmatic research perspective that often interprets violent behaviors through a moral lens. A deeper investigation into verbal aggression, guided by affective pragmatics, acknowledges the role of affect and facilitates a more accurate interpretation of intentions based on emotions, thereby enriching our understanding of human communicative behaviors by taking affect parameters into account and enabling the deduction of intentions based on emotions. Besides, prosodic features like pitch, duration, and intensity could contribute to emotion recognition and the pragmatic interpretation of emotional expressions. Saeed and Ashraf (2023) utilized the Hidden Markov Model (HMM) as a quantitative data and prosodic modeling tool to analyze emotional recordings and enhance the understanding of affective pragmatics, which provides deeper insights into the intersection of emotional prosody and pragmatic meaning.

In conclusion, affective pragmatic behavior is an inescapable phenomenon, deeply rooted in the psychological and biological characteristics of humans. As research progresses, our understanding of individuals’ affective pragmatics will deepen, making the theory of affective pragmatics a vital supplement to existing pragmatic theories.

### 2.2 Affective pragmatics belongs to Darwinian biolinguistics

The affinity between affective pragmatics and Darwinian biolinguistics could be traced back to the foundational theories of Charles Darwin. Darwin posited that the evolutionary trajectory of the human species led to the dispersion of various tribes across different geographical landscapes, resulting in notable diversifications in physical characteristics such as skin colour, hair texture and facial features, as well as temperamental and esthetic inclinations. He further articulated that the inherent sexual, parental, and social instincts in humans form the biological underpinnings of moral emotions (Darwin, 1871). This proposition implies that prior to the development of language, humans likely experienced emotional responses to their environment. Moreover, Darwin suggested that the lives of early humans were predominantly driven by instinctual desires over rational deliberation, a behavior pattern that has been perpetuated through evolutionary inheritance (Darwin, 1871). This historical perspective underscores the significance of understanding the role of innate biological factors in shaping affective responses and highlights the evolutionary continuity present in human affective pragmatics. Infants start to express unconscious emotions through specific facial expressions caused by muscle movements of the facial features (eyebrows, eyes, ears, nose, lips) early on. Gradually, a habitual association among specific situational stimuli, emotions, and behaviors will be established, such as trembling triggered by fear from a danger signal (e.g., Darwin, 1872; Watson and Rayner, 1920). Whenever a person encounters a stimulus that evokes emotions and prompts action plans, their nervous and muscular systems must adapt and prepare, resulting in visible expressions. Genuine expressions are the outcome of a holistic response involving the animal’s internal organs, nervous system, and facial muscles, distinctly contrasting with expressions rooted in learned, insincere emotions like a forced smile (Darwin, 1872). This perspective of the natural response mechanism of emotions lays the biological theoretical foundation for affective pragmatics to infer pragmatic intentions from emotional signals.

Biologically, human desires and emotions stem from numerous processes within the cerebral cortex. Positive emotions emerge when the processes of excitation and inhibition within the cerebral cortex proceed smoothly, whereas negative emotions (such as fear and timidity) arise when these processes are disrupted (Jacobson, 1988:102). This mechanism ensures that the human brain generates precise and consistent responses to the external environment, including societal stimuli. Human emotions also produce complex responses as the context changes (Jacobson, 1988:111). In alignment with Darwin’s perspective, Basic Emotion Theory posits a direct correlation between emotions and brain neurons, proposing that each emotion is governed by a unique neural circuitry, distinctively separate from those of other emotions. Furthermore, it suggests that human genes encapsulate a fundamental emotional defense system (Ekman, 1992). It’s proved by experimental data that emotion is the result of a combination of synaptic connections, cell interactions and electrical responses, as the result of embodied cognition, confirming that emotions are situated between reactions and consciousness in a pre-linguistic stage between stimulus and behavior (Papoulias and Callard, 2010). Advancements in the fields of emotion research and neuroscience, particularly through the application of fMRI brain imaging, have substantiated the theory by identifying specific neural correlates of emotions. Notably, there is a demonstrable mapping between fear and the amygdala, sadness and the anterior cingulate gyrus, anger and the orbitofrontal cortex, as well as disgust and the insula (Lindquist et al., 2012). This empirical evidence reinforces the foundational premise of Basic Emotion Theory by elucidating the neural substrates underpinning distinct emotional responses. It can be understood that external stimuli will trigger an individual’s emotional neural response, which is then displayed as speech acts and paralinguistic signals in communication.

These discussions have established the foundation for the field of affective pragmatics and Darwinian biolinguistics, which scrutinizes the evolution of language from a biological perspective. Pennisi and Falzone (2016) have argued that pragmatics can be seen as a biolinguistics of performance, suggesting that the way language is used in real-life situations can be studied through the lens of biological evolution. Because pragmatics, different from syntax and semantics, refers to the use of language in context, including how speakers understand and convey meaning based on the situation, Pragmatic intentions behind the speech acts and the inference of meanings are usually not explicitly stated. This research perspective aligns with the Darwinian approach to biolinguistics, which views language not just as a system of communication but as a complex, adaptive system that has evolved to meet the communicative and social needs of humans. Thus, it suggests that to fully understand the nature of language, one must consider not only its structural aspects but also its functional and adaptive roles in human life. This holistic approach has expanded the field of linguistics to include insights from biology, anthropology, and psychology, enriching our understanding of language as a fundamental aspect of human nature and evolution.

Furthermore, the physiological and psychological foundations of affective pragmatics are deeply intertwined with the biological traits of individuals, which are not only capable of evolution but also subject to dynamic changes. Building on the factors previously mentioned, the exploration of affective pragmatics is intrinsically connected to Darwinian biolinguistics, embodying an evolutionary viewpoint on the significance of language in human cognition and communication. In other words, affective pragmatics and Darwinian biolinguistics converge on the essential role of language in facilitating cognitive processes and thought. “Pragmatic approach to a cultural paradigm depends on the way we understand cognitive intentionality”(Pennisi and Falzone, 2016:258).

Moreover, individual cognitive intentionality is directly linked to biolinguistics, or more accurately, to Darwinian biolinguistics. “Emotions, feelings, and other mental states, in fact, constitute cognitive pre-conditions for any type of expression”(Pennisi and Falzone, 2016:65). “They may have played an initial role to enable increasingly complex cognitive functions, performing in different communication modalities” (*ibid*). These elements likely played a crucial role in the evolution of complex cognitive functions and diverse modes of communication. Additionally, affective pragmatics highlights the profound impact of emotion on cognition and speech acts, underscoring that research in this area should commence with the identification of individuals’ emotions. However, emotions, being internal human activities, prove challenging to monitor. Biologically, unconscious emotional physiological responses include changes in the sympathetic and parasympathetic nervous systems, such as dilation of skin capillaries, trembling, sweating, shrinking or bulging of the forehead veins, blushing, deep or shallow breathing, or pupil dilation (Scarantino, 2017a,b). Individuals’ biological parameters could facilitate the interpretation of internal emotions but depend on a specific amygdala neural pathway in the brain for recognizing these signals. If this pathway is interrupted or damaged, people’s ability to recognize and distinguish facial expressions will decrease (Evans, 2002). Therefore, both the production and identification of emotions are closely linked to individuals’ biological attributes. Current EEG techniques are capable of monitoring brain activity associated with both positive and negative emotions, as demonstrated by studies (e.g., Prete et al., 2022). This implies that the tools used in biolinguistic research are beneficial for studies in affective pragmatics. Given the intertwined nature of emotion and cognition, where emotion is considered an integral component of cognition (Wharton and de Saussure, 2024), biological data collected by EEG or fMRI technology can yield scientific evidence allowing us to observe participants’ emotional fluctuations and cognitive pathways, thereby offering profound insights into the intentions behind their speech acts.

## 3 Examples of affective pragmatic analysis

Affective pragmatics recognizes that language serves not just as a conduit for conveying factual information but also as a potent means for expressing and invoking emotions. In the realm of practical communication, even individuals renowned for their rationality and reason can, at times, express themselves in ways that seem irrational, driven by strong emotions. For instance, Oscar Wilde, the celebrated Irish playwright and writer, penned a notable letter voicing his indignation at societal injustice and suffering. This letter, famously known as the ‘De Profundis’ letter, serves as a poignant example of emotion-driven communication. Similarly, Donald Trump, a figure of political leadership, often made statements and took actions that many viewed as surpassing the rational expectations typically held for political figures. These instances underscore the reality that individuals, regardless of their status or achievements, are capable of displaying behavior that deviates from rational norms, influenced by intense emotional states, leading to speech acts displaying irrational reasoning.

There, it is essential to consider not merely the explicit meanings of words but also the contextual, cultural, and personal elements that shape the emotional resonance and reception of messages. The application of affective pragmatics to understand those real-world situations is particularly revealing. With illustrative examples, the dynamic essence of affective pragmatics is brought to the forefront, providing deep insights into the emotional currents that permeate everyday exchanges. In so doing, one should bear in mind the basics that are affective pragmatics, including its core assumption, new understanding of emotional expressions, and its basic tenets and how they are applied to judging the pragmatic results of the specific communication events.

The core proposal of affective pragmatics is that “emotional expressions are a means of expressing inner states, of representing what the world is like, of directing other people’s behavior, and of committing to future courses of action” (Scarantino, 2019: 49). Meanwhile, emotion can be expressed by either verbal forms or nonverbal forms or both, dependent on context. This proposal differs affective pragmatics from that of the Gricean tradition which prioritizes interrogating what people infer from what is verbally conveyed to them (Grice, 1957; Sperber and Wilson, 1995; Levenson, 2000) and ignores the nonpropositional meaning in interpersonal communication, that is expressed beyond words (attitudes, beliefs, emotions, etc.), whose primary and possibly most efficient medium is nonverbal language and which is also parasitic on and omnipresent in verbal language.

**Figure 2:**
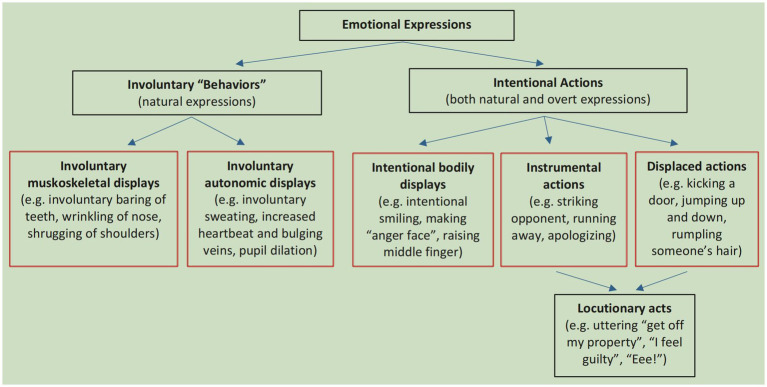
Five main ways to express emotions. Reproduced with permission from Scarantino (2019: 60).

Emotional expressions include overt emotional expression and natural emotional expression. The former means “A sequence of bodily changes X of some agent A in context C overtly expresses A’s emotion E relative to recipient R insofar as X was produced by A to make R infer that A is experiencing E while making A’s intention manifest to R” (Scarantino, 2019: 51), the latter means “A sequence of bodily changes X of some agent A in context C naturally expresses A’s emotion E relative to recipient R insofar as X increases the probability that A is undergoing E, relative to R’s background knowledge” (*ibid*). Both can be produced in either verbal form or nonverbal form; both can be produced voluntarily or involuntarily; both can carry either natural or non-natural information. Taking all the above considerations, emotional expression can be further divided into five sub-types, as follows (Figure 2).

Emotional expressions, whether in verbal forms or nonverbal forms, perform speech act analogs that produce communicative effects. Specifically, with emotional expressions, interlocutors involve themselves in communicative moves that are analogous to such illocutionary acts as follows:

Expressives_EE_ have the communicative point of expressing the signaler’s emotions by means of natural or non-natural information transfer. Commissives_EE_ have the communicative point of committing the signaler to a future course of action by means of natural or non-natural information transfer. Imperatives_EE_ have the communicative point of trying to get the recipient to do something by means of natural or non-natural information transfer. Declaratives_EE_ have the communicative point of representing how things are in the world by means of natural or non-natural information transfer. (Scarantino, 2019: 67)

Summarily, the above core assumptions, the classification of emotional expressions, and the main tenets are essential for people to understand affective pragmatics. On the other hand, they are the principles and guides that people can adopt to examine the role emotional expressions play and to grasp the differences among rational, emotional, and neutral speech acts in specific communication events, as demonstrated by the examples provided below.

Here, the rational and neutral dialogues are meticulously crafted to resemble everyday conversations, while the emotional dialogue is derived from a drama in presidential debate.

1. Rational:

*Context*: A scientific conference on climate change.

*Participants*: Dr. Alice (a climate scientist) and Dr. Bob (an environmental policy expert).

*Dialogue:*

Dr. Alice: *Based on the data we have collected, there is a 90% likelihood that temperature increases are significantly influenced by human activities. Our models predict a 2-degree Celsius rise by 2050.*

Dr. Bob: *That aligns with policy recommendations. To mitigate this, global emissions must be reduced by 40% in the next decade. What are your thoughts on implementing carbon capture technology?*

Dr. Alice: *It’s a viable solution but requires international co-operation and substantial investment. We should also consider the socioeconomic impacts.*

Predominantly, this is a verbal communication in which participants engage in discussions centered around data, scientific prognostications, and the policy implications. Both sides depend on data and policy implications for arguments, with no overt emotions being expressed. They perform Declaratives_EE_ that have the communicative point of representing how things are in the world using natural or non-natural information transfer. Their ultimate purpose is to exchange ideas and reach an agreement on the issue in question.

In this rational scenario, belonging to CAV or CVA type, both participants adhere to the four cooperative principles, allowing their intentions to be effortlessly inferred from the literal meaning of their words. Despite its rational nature, this dialogue can still be analyzed through the lens of affective pragmatics to elucidate their underlying pragmatic intentions. To be specific, Dr. Alice’s initial statement exemplifies her intent to disseminate scientific discoveries, showcasing a comprehensive grasp of the data and its ramifications. The tone is neutral—factual and stripped of personal emotion, categorizing it as a rational speech act dedicated to the dissemination of scientific knowledge, firmly anchored in empirical evidence. In contrast, Dr. Bob’s reply manifests his ambition to bridge the gap between scientific insights and policy formulation. He not only grasps the significance of the data but also contemplates actionable steps (policy suggestions), focusing on policy implications rather than personal sentiments. This, too, is a rational speech act, with Dr. Bob zeroing in on the pragmatic application of data within the sphere of policy-making. Dr. Alice’s subsequent comment reveals her keenness to voice her perspective on a viable solution. Her familiarity with carbon capture technology and its prerequisites is evident.

A subtle hint of caution or concern betrays a slight emotional undercurrent, reflecting apprehensions about the solution’s practicality and broader societal impacts. Though this speech act is predominantly rational, concentrating on the logistical execution of a solution, it encompasses an emotional dimension as it navigates the societal repercussions and underscores the necessity for collective action. In all, this dialogue is to maintain a rational demeanor. A latent concern for the future permeates their exchange, albeit viewed through a rational prism. The employment of specialized terminology and the citation of concrete data and policy measures reveal an elevated level of cognitive engagement. Their shared determination to tackle climate change and investigate potential remedies is apparent, showcasing volition. Emotional elements are restrained yet detectable, especially in Dr. Alice’s reflections on the socio-economic consequences, introducing a human element into the scientific discourse.

To encapsulate, the discourse is chiefly rational, motivated by an earnest endeavor to comprehend and mitigate a critical issue, subtly laced with emotional undertones regarding the consequences of their deliberations and suggestions. Consequently, both speakers exhibit a composed psychological demeanor with no pronounced biological manifestations, rendering their discourse predominantly rational.

1. Emotional:

*Context*: parody show of the presidential campaign debate.

*Participants*: Trump and Clinton.

*Dialogue*:

Trump: *They are ripping babies out of vaginas* (pointing finger in the air).

Clinton: (Opens her mouth, widens her eyes, and shakes her head) *Chris. I’m glad you raised this topic*, *because what better people are there to talk about women’s issues*? *Me, a woman who has had a child and has taken birth control*, *and him, a man who is a child and whose face is birth control*.

Trump: (Shakes his head, opens his mouth, leans forward).

The above dialogue is a clip taken from a parody show called SNL (Saturday Night Live). The actors’ performance serves as a quintessential example of ACV or AVC type, and should be best analyzed through the lens of affective pragmatics. Both participants notably deviate from the four cooperative maxims of Gricean pragmatics as well as from established politeness principles. The two participants are involved in verbal as well as nonverbal forms of communication. Both sides scratch their brain to present evidences for their arguments, with overt emotional expressions, voluntary and involuntary, being used to help. They perform the Declaratives_EE_ which have the communicative point of representing how things are in the world using natural or non-natural information transfer and the Expressives_EE_ which have the communicative point of expressing the signaler’s emotions by means of natural or non-natural information transfer. Their ultimate purpose is to struggle against each other and to win support from the voters.

Particularly, this debate differs strikingly in emotional expression from the previous rational discussion. As illustrated in the debate, when emotions surpass the threshold, interlocutors’ biological state becomes the signal to deliver additional cues of intention. Trump, articulating his stance on abortion from a position of anger, described it as inhumane, particularly criticizing late-term procedures. Clinton, taken aback by Trump’s assertions, showcased her disagreement through verbal and nonverbal cues including open-mouthed stares and head shakes. Embracing the opportunity to address the topic, Clinton appeared to leverage it to underscore her policy strengths and connect with voters, positioning herself as uniquely qualified to discuss the issue due to her personal and professional experiences related to childbirth and contraception. Clinton’s strategy seemed to pivot on showcasing her distinct perspective as a woman, aiming to resonate with voters on a deeply personal level. She attempted to cast Trump’s views in a negative light, suggesting they were out of touch and offensive, which she punctuated with humor aimed at his expense, suggesting his opinions were as ineffective as “failed birth control.” This tactic appeared designed to provoke frustration in Trump, who responded with his own set of nonverbal cues that included mouth opening, head shaking, and leaning forward, perhaps to maintain composure and project a respectful demeanor despite the heated exchange. The debate highlights not just the content of what was said but the complex interplay of emotion, gender dynamics, and political strategy, revealing how both candidates sought to navigate the highly charged atmosphere and connect with the electorate.

The natural and visible physical reactions to intense emotions, such as those displayed by actors through their expressions, body language, and gazes, underscore the physical manifestations of their emotional states. Given that “affect is a part of cognition” (Wharton and de Saussure, 2024), the speech acts of both participants were significantly influenced by their strong emotions. The evident incivility between them during the debate exposed their genuine perceptions of each other under the influence of those emotions. Thus, affective pragmatics takes the view that emotions, when uncontrolled, can escalate to provocation, abuse, or even violence, inflicting both physical and psychological damage.

1. Neutral:

*Context*: A casual conversation between two colleagues during lunch break.

*Participants*: Sarah (a graphic designer) and Mark (an IT specialist).

*Dialogue*:

Sarah: *I saw the forecast; it might rain later this afternoon.*

Mark: *Oh, really? I guess I should have brought my umbrella. Thanks for letting me know.*

Sarah: *No problem. Have you tried the new coffee machine in the break room?*

Mark: *Not yet, but I heard it’s good. I’ll give it a try after lunch.*

The aforementioned neutral dialogue, mostly belonging to VCA or VAC type, represents typical lunchtime conversations among office workers, occurring almost daily. In these exchanges, they discuss everyday tasks and mundane facts in an informal manner, without the explicit expression of emotions. Both participants mainly observe the cooperative principles and politeness principles. Thus it could be explained from the Gricean pragmatics or affective pragmatics. Overall, both participants perform Declaratives_EE_ that have the communicative point of representing how things are in the world by means of natural or non-natural information transfer. Their ultimate purpose is to maintain a smooth and friendly relationship in the workplace.

In specific, Sarah’s first statement displays her wish to share useful information about the weather. She is aware of the weather forecast and its potential impact. Her statement is neutral, as it conveys information without emotional emphasis. This is a rational and neutral speech act. Sarah is sharing information that could be useful for planning the day, without any emotional overtones. As to Mark’s response, he expresses a realization and appreciation for the information. He acknowledges the usefulness of the information and its practical implication (carrying an umbrella). Mark’s speech act is rational and neutral. He recognizes the utility of the information and expresses a mild regret for not being prepared, but no strong emotional content is present. Sarah’s second statement aims to shift the conversation to another topic of potential interest. She is aware of changes in the workplace environment (new coffee machine). The statement is neutral, aiming to engage in light and friendly conversation, serving to continue the conversation, and potentially deepening the social connection with a colleague. Mark’s second response expresses his want to respond to the new topic and maintain the conversation. He knew about the coffee machine and expresses his intent to try it. This response is also neutral, indicative of casual workplace dialogue. Mark’s speech act is neutral and slightly rational, focusing on his intention to try the new machine based on its reported quality.

Overall, both speakers are engaging in a typical workplace conversation that is more informational and practical than emotional. Their volition in this context is about sharing information and maintaining a pleasant conversation. Cognition is evident as both Sarah and Mark are aware of their environment (weather, workplace amenities) and its implications. Affect plays a minimal role, as the conversation remains in the realm of casual workplace small talk without delving into personal or emotional topics. Thus, it can be inferred that both of them did not have apparent physical reactions; their dialogues just revolved around sharing useful information in an everyday, common conversation.

Such everyday conversations, as seen in examples 1 and 3, are typically categorized as either rational or neutral and have traditionally been analyzed through the lens of Gricean pragmatics, which elucidates how participants’ speech acts adhere to cooperative principles. However, through psychological analysis, affective pragmatics goes a step further, not only illustrating participants’ intentions but also shedding light on the reasons behind their rational, neutral, or emotional responses. This approach offers a more detailed insight into the underlying logic of their behavior, because affective pragmatics views emotional acts as catalysts for actions, as posited by Scarantino (2017a,b).

Nevertheless, this perspective raises certain questions. One may wonder why individuals continue to engage in rational and neutral speech acts. The psychological concept of a ‘threshold’ offers an explanation. This is because, for an emotional response to influence behavior, it must exceed a specific threshold. This affective threshold varies among individuals and is influenced by a range of factors, including cultural background, personal experiences, and psychological state. To comprehend affective thresholds, one can imagine a scale measuring emotional intensity. When emotions are subdued, an individual’s speech typically remains rational, unaffected by these minimal emotional states. However, as emotional intensity crosses this threshold, it starts to impact cognition, resulting in speech acts that are emotional or imbued with affect. Therefore, rational speech acts, characterized by logic, clarity, and objectivity, prevail when emotional intensity lies below this threshold. In contrast, when emotional intensity exists but does not reach extreme highs or lows, individuals are likely to produce neutral speech acts—that are neither overtly rational nor emotional. It’s essential to recognize that individuals with a lower affective threshold might respond with emotional speech acts to relatively mild emotional triggers, whereas those with a higher threshold might continue to exhibit rational speech patterns, even in contexts with more pronounced emotional undercurrents. The way emotions are expressed and interpreted is significantly shaped by cultural norms, which in turn affect these thresholds. As a result, understanding the impact of cultural and contextual factors is key to comprehending these thresholds and their effect on speech acts. Those emotional ones are the focus of affective pragmatics and bio-linguistics.

Delving into the nexus between affective thresholds and speech acts unveils deeper insights into the dynamics of affective pragmatics. When an individual’s emotional intensity skyrockets, it may even lead to disordered speech patterns. A common subject within affective pragmatics is stuttering. Moreover, as a complex phenomenon influenced by genetic, developmental, and environmental factors, it occupies a central place in the field of biological linguistics, as highlighted by scholars such as Benítez-Burraco (2023). Consider the case of King George VI, who found himself thrust onto the throne following his brother’s abdication. This unexpected rise to monarchy brought with it an enormous weight of responsibility, compounded by his struggle with a stammer—a challenge magnified under the public and personal pressures of his new role. King George VI serves as a quintessential example. It’s a pity we do not have direct EGG or fMRI data on him, but it is plausible to infer that the emotional stress and anxiety associated with the overwhelming demands of his role aggravated his speech impediment. This scenario underscores the profound influence emotional states can exert on speech capabilities. Affective pragmatics posits that our emotions significantly shape our communicative methods. In instances of emotional disturbances, these influences can manifest as notable disruptions in regular speech patterns, emphasizing the intricate interplay between emotional well-being and linguistic expression. It’s reasonable to assert that stress and anxiety can exacerbate the neurological and cognitive disturbances associated with stuttering, leading to exacerbated speech impediments. This situation often results in a vicious cycle, where the dread of speaking and ensuing speech difficulties feed into each other, further aggravating the condition. The complex interrelation between emotional states and language disorders serves as a poignant illustration of the critical juncture between affective disorders and linguistic challenges. This case highlights the necessity of adopting a comprehensive approach toward language and emotional health, acknowledging the interconnectedness of these spheres in both clinical and historical settings.

The common biological foundations of emotional speech acts linking the theoretical and practical aspects of affective pragmatics and biolinguistics hinting at a conjoined future for these fields. Thus, exploring affective pragmatics may shed light on the mechanisms underlying atypical speech acts in bio-linguistics, providing insightful perspectives on the complex interplay between emotional states and language disorders.

## 4 Queries of affective pragmatics and its shared prospects with biolinguistics

Although affective pragmatics has complemented rationalist pragmatics by taking affect into the analysis of daily communication, it has faced great challenges (Scarantino, 2017b). One of the oppositions is that paralinguistic behaviors like facial expressions cannot perform a declarative function as verbal codes do, so they cannot fully replace the communicative function of language (Fischer, 2017). This question could be answered by Darwinian biolinguistics, as “human cries and bodily symbols, such as emotional signals, are the origin of language and the basis of cognition and communication” (Darwin, 1872). So nonverbal language is the first language. The para-languages cannot perform directly a declarative function, but they convey subconsciously more important information related to identity, attitude, stance, etc. Besides, behaviors such as body orientation, standing position, and eye contact suggest people’s subconscious psychological distance from and focus on events. Indeed, beyond paralanguage, affective pragmatics leverages the speaker’s speech behaviors to discern emotions and deduce intentions. The intricate nature of emotions and technical constraints distinguish the research methodologies of affective pragmatics from those of traditional pragmatics.

In fact, affective pragmatics aligns with Carnap’s philosophy, emphasizing the decoding of natural meanings embedded in emotional signals (Scarantino, 2017b:218). It’s a project of Carnapian pragmatics rather than Gricean pragmatics (*ibid*:219). Proponents argue that the meaning of discourse ought to be inferred from psychological constructs, advocating for an in-depth examination of the internal emotional states of individuals engaged in communication, which is also the argument of Post-Gricean pragmatics.

Affective pragmatics also incorporates into its research insights from psychology and neuroscience. They believe all affective phenomena are experientially and mechanistically organized from the perspective of the human organism (Schiller et al., 2023: 31). Tools like fMRI scans have been instrumental in linking specific emotions to brain activity, further solidifying the biological basis of this field. Empirical studies in linguistics and real-world observations help people to understand better how emotions influence language in diverse cultural and social contexts. The analysis of affect offers valuable insights into interpersonal relationships, enhancing our understanding of empathy, conflict resolution, and emotional intelligence, especially in cross-cultural communication where interpreting emotional nuances in different cultural contexts is vital.

Thus, emotional signals, subjects’ verbal thoughts, and researchers’ data analysis of subjects’ speech behaviors can form a triangulation of data verification for affective pragmatic research. Although current applications of affective pragmatics are limited, European scholars have detected, through EGG devices in the laboratory, that subjects produce corresponding ERP potentials in the brain when faced with words carrying different emotional loads, indicating that the evoked emotional responses affect subsequent pragmatic behaviors (Jończyk, 2016). Since neurolinguistics can provide empirical data for affective pragmatics research, their integration can give rise to a new research direction of Affective Neuro-Pragmatics (*ibid*). Recent studies show that different speech acts, such as requests or apologies, activate different brain regions, and constructing pragmatic models based on the correlation between numerous speech acts and neural potentials is a direction actively explored in academia (Boux et al., 2021). The process of attempting to construct affective pragmatic models can enhance the understanding, tolerance, and control of affective-cognitive-volitional or affective-volitional-cognitive behaviors, promoting social harmony.

Now more and more researchers (e.g., Ding and Zhang, 2023) have noticed the role that emotion plays in language disorders, which means affective pragmatics could help language education and therapy. The principles of affective pragmatics may help educators and therapists to better address the emotional aspects of language learning or language disorders. Also, it may help to inform the development of artificial intelligence, particularly in creating more emotionally intelligent and responsive communication technologies.

Biological linguistics explores the brain regions and neural pathways involved in language processing. Affective pragmatics is concerned with how emotional meaning is conveyed and interpreted in language. By studying the neural correlates of both language and emotion, researchers can understand how the brain integrates emotional content into linguistic expressions. The prefrontal cortex, known for its role in emotion regulation, is also involved in language processing. Biological linguistics can reveal how this area of the brain helps manage emotional responses during communication, a key interest of affective pragmatics. From an evolutionary perspective, biological linguistics examines how language evolved as a cognitive faculty in humans. Affective pragmatics can benefit from this by revealing how the evolution of language included the capacity for emotional expression and understanding, essential for social bonding and survival since some biological research (e.g., Benítez-Burraco, 2023) delves into language disorders and co-occurrences of language and emotion (e.g., Toseeb et al., 2023). For example, in autism, the ‘so-called “contextual information” is tightly linked to people’s affective dispositions, how the meanings retrieved are often less than a research topic of fully determinate propositions and how these testable predictions help to understand pragmatic language difficulties have only begun to be explored’(Ifantidou and Wharton, 2024:35). Thus, affective pragmatics may provide clues about language disorders, or even insights into the roots of the problems, bridging biological linguistics with affective pragmatics. Biological linguistics can illustrate the biological causes of language disorders, while affective pragmatics can extend this to explore how these disorders influence emotional aspects of language, which, in turn, cause language disorders. Similarly, insights into the biological bases of emotional dysregulation can inform affective pragmatics, particularly in how such dysregulation impacts language use and interpretation.

In conclusion, the symbiotic and multidimensional relationship between affective pragmatics and biolinguistics underpins significant advancements in understanding the interplay between emotion and language. Biolinguistics lays the groundwork by elucidating the brain’s language capabilities and genetic foundations, essential for affective pragmatics to investigate the encoding, processing, and comprehension of emotional content in language. This interdisciplinary approach not only propels theoretical knowledge forward but also bears practical implications in fields such as therapy for communication disorders, artificial language processing, and the analysis of social interaction dynamics.

The integration of affective pragmatics with new technologies and biological linguistics is poised for a dynamic and promising future. The collaboration between linguists, neuroscientists, and psychologists is expected to become increasingly prevalent, fostering holistic studies on the nexus of emotion and language. Insights from affective pragmatics are set to translate into tangible benefits, enhancing communication strategies for individuals with emotional or language impairments. The advent of wearable technology for the real-time tracking of physiological responses during communication promises to deliver concrete data, enabling the exploration of emotions’ impact on language in naturalistic settings. Researchers can also use natural language processing tools like Python to automatically extract and analyze emotional clues in large text datasets. Such methods have proved to successfully identify emotional disorders. It’s a matter that Theory of Affective Pragmatics and Biolignsuitics could cooperate, and their collaboration will have a promising future.

Anticipating future research in affective pragmatics, it’s clear that technological progress and a deeper understanding of biolinguistics will significantly influence the field. These advancements will facilitate a comprehensive grasp of the complex relationship between affect and language, potentially revolutionizing theoretical perspectives and practical applications across linguistics, psychology, and communication technologies. The convergence of theoretical and practical aspects of affective pragmatics and biolinguistics is expected to uncover profound insights into how emotions shape language usage and comprehension, heralding a new era of interdisciplinary research and application.

## 5 Conclusion

This paper provides a succinct overview of the evolution of affective pragmatics, illustrating the indissoluble link between biological constitution and linguistic capabilities. It highlights how affective pragmatics, drawing inspiration from Darwinist biolinguistics, promises fresh insights into the pragmatic meanings of affect—insights that rationalist approaches often overlook. Indeed, emotional expressions transcend simple linguistic constructs; they are deeply enmeshed with our biological core, simultaneously influencing and being shaped by our cognitive processes.

Despite the challenges affective pragmatics faces in interpreting and categorizing the complexity of human emotions, it opens up novel avenues for investigating the interplay between language and emotion in communication. With technological advancements, affective pragmatics is poised to shed light on a broader range of communication issues, captivating the interest of linguists, psychologists, physiologists and cognitive scientists alike.

In conclusion, this paper advocates for a holistic approach to pragmatics. Adopting a biolinguistic viewpoint not only deepens our comprehension of affective pragmatics but also unveils new pathways for research in the domains of language, communication, and cognitive science.

## Data availability statement

The raw data supporting the conclusions of this article will be made available by the authors, without undue reservation.

## Ethics statement

Written informed consent was obtained from the individual(s) for the publication of any potentially identifiable images or data included in this article.

## Author contributions

LZ: Writing – review & editing, Writing – original draft, Visualization, Validation, Supervision, Software, Resources, Project administration, Methodology, Investigation, Funding acquisition, Formal analysis, Data curation, Conceptualization.
